# Comparing Self-reported Disease Outcomes, Diet, and Lifestyles in a National Cohort of Black and White Seventh-day Adventists

**Published:** 2007-06-15

**Authors:** Susanne Montgomery, Patti Herring, Larry Beeson, Terry Butler, Synnove Knutsen, Joan Sabate, Jacqueline Chan, Gary Fraser, Antronette Yancey, Susan Preston-Martin

**Affiliations:** Loma Linda University, School of Public Health; Loma Linda University, School of Public Health, Loma Linda, Calif; Loma Linda University, School of Public Health, Loma Linda, Calif; Loma Linda University, School of Public Health, Loma Linda, Calif; Loma Linda University, School of Public Health, Loma Linda, Calif; Loma Linda University, School of Public Health, Loma Linda, Calif; Loma Linda University, School of Public Health, Loma Linda, Calif; Loma Linda University, School of Public Health, Loma Linda, Calif; University of California at Los Angeles, School of Public Health, Los Angeles, Calif; University of Southern California, Department of Preventive Medicine, Los Angeles, Calif

## Abstract

**Introduction:**

Few epidemiologic cohort studies on the etiology of chronic disease are powerful enough to distinguish racial and ethnic determinants from socioeconomic determinants of health behaviors and observed disease patterns. The Adventist Health Study-2 (AHS-2), with its large number of respondents and the variation in lifestyles of its target populations, promises to shed light on these issues. This paper focuses on some preliminary baseline analyses of responses from the first group of participants recruited for AHS-2.

**Methods:**

We administered a validated and pilot-tested questionnaire on various lifestyle practices and health outcomes to 56,754 respondents to AHS-2, comprising 14,376 non-Hispanic blacks and 42,378 non-Hispanic whites. We analyzed cross-sectional baseline data adjusted for age and sex and performed logistic regressions to test differences between responses from the two racial groups.

**Results:**

In this Seventh-day Adventist (Adventist) cohort, blacks were less likely than whites to be lifelong vegetarians and more likely to be overweight or obese. Exercise levels were lower for blacks than for whites, but blacks were as likely as whites not to currently smoke or drink. Blacks reported higher rates of hypertension and diabetes than did whites but lower rates of high serum cholesterol, myocardial infarction, emphysema, and all cancers. After we eliminated skin cancer from the analysis, the age-adjusted prevalence of cancer remained significantly lower for black than for white women. The prevalence of prostate cancer was 47% higher for black men than for white men.

**Conclusion:**

The profile of health habits for black Adventists is better than that for blacks nationally. Given the intractable nature of many other contributors to health disparities, including racism, housing segregation, employment discrimination, limited educational opportunity, and poorer health care, the relative advantage for blacks of the Adventist lifestyle may hold promise for helping to close the gap in health status between blacks and whites nationally.

## Introduction

The influence of lifestyle factors such as diet and physical activity and of genetic and metabolic predisposition on the incidence and progression of chronic diseases is well established (1,2). Findings on the independent effects of diet and physical activity on disease outcomes, however, are conflicting. Experts suggest that these differences lie in a lack of precision in estimating the effect of small ranges of exposures and in distortions due to measurement errors (3). Furthermore, few epidemiologic cohort studies on the etiology of chronic diseases are powerful enough to distinguish racial and ethnic determinants from socioeconomic determinants of health behaviors and disease patterns.

To help shed light on these issues, a longitudinal study is needed that recruits sufficient numbers of black* and white respondents to provide subsamples for disease endpoints that are large enough to create prediction models that include lifestyle factors. The population in such a cohort should have significantly varied dietary and other lifestyle exposures, and the study should use methods of analysis that take the challenges of this type of study into account. The Adventist Health Study-2 (AHS-2), with its anticipated 30,000 black and 70,000 non-Hispanic white Seventh-day Adventist (Adventist) participants is one such study. The study's design and large numbers, the variability in lifestyles of its target populations, and the proposed novel analytic methods are promising. This paper focuses on some preliminary baseline analyses of responses from the first 56,754 respondents recruited for AHS-2. Recruitment of the balance of the targeted 100,000 participants is in progress.

*The term *black* is used because the Adventist population includes descendents of African American slaves, people of Afro-Caribbean background, and more recent immigrants from a number of African countries.

Adventists are a Christian Protestant denomination that counsels its members to avoid alcohol, tobacco, and pork and recommends a vegetarian diet. Church members follow these recommendations to varying degrees. The National Institutes of Health funded two previous longitudinal studies of Adventists in California: the Adventist Mortality Study (AMS) (4,5) from 1960 to 1966 and the first Adventist Health Study (AHS-1) (6,7) from 1974 to 1988. These studies indicated that the risk for cardiovascular disease, diabetes, and most cancers was lower for Adventists than for non-Adventists and that life span was 7.3 years longer for Adventist men and 4.4 years longer for Adventist women than for men and women in the general California population (7).

About 35% of Adventists who participated in AHS-1 were lacto-ovo vegetarian (i.e., ate dairy and eggs), 3% were vegan (i.e., ate no meat, dairy, or eggs), 20% ate meat less than once a week, and the remainder ate meat at rates similar to those of non-Adventists (7). This population also has low levels of smoking (1.8%) and alcohol consumption (less than 10%) (2), practices that have been major confounders in the epidemiologic study of chronic disease. Furthermore, analysis of three 24-hour recalls from approximately 350 AHS-2 subjects in a calibration substudy found that their mean exercise levels were 50% higher than the U.S. national average but showed great individual variation. Forty-five percent ate soy protein at levels similar to those seen in China and Singapore (8,9; AHS-2, unpublished data, 2006).

A number of studies indicate that Adventists have health advantages over the general, non-Adventist population (4-6,10,11). The study that first identified the relationship between vegetarianism and the prevention of type 2 diabetes, heart disease, cancer, and overall mortality involved an Adventist population (2,12). The California study showed the prevalence of diabetes to be approximately 100% higher for non-vegetarian than for vegetarian Adventists and the prevalence of hypertension to be approximately 200% higher (7). This study also found that vegetarianism, nut consumption, normal body mass index (BMI), physical activity, and not smoking contributed substantially to the observed increase in life expectancy for Adventists over other Californians (7). Because these studies lack sufficient numbers of black respondents, however, it is unclear if and how their findings translate to black Adventists. Answering these questions could add significantly to our understanding of the relationship of lifestyle and genetics to the etiology of chronic diseases.

Heart disease, the leading cause of death in the United States (13), affects blacks disproportionately. Overall, the incidence of cardiovascular disease (CVD) is higher for black than for white women, and mortality from CVD and hypertension is higher for blacks than for whites of both sexes (14,15). For AHS-1 participants with a follow-up of 12 years, however, rates of these three conditions were considerably lower than those for blacks nationally (16). Physical activity; absence of red meat in the diet; consumption of nuts, fruits, vegetables, and whole grains; and sufficient water consumption were associated with this lower risk. Given the limited sample size of the original study, however, the degree of benefit from these factors is clearer for whites than for blacks.

Rates of morbidity and mortality from cancer, the second leading cause of death in the United States, are also generally higher for blacks than for whites (17,18), and cancer survival rates are lower for blacks than for whites for almost all cancers regardless of site and stage (18,19). Although evidence that fruit and vegetable consumption protects against many cancers is convincing (2,20), results on the effects of specific nutrients and dietary patterns such as vegetarianism remain contentious and differ by type of cancer (4-6,10,11,20-25). We expect that AHS-2, with its larger black population and pertinent information on lifestyle factors, will help elucidate the degree of protection from heart disease, cancer, and other chronic diseases that lifestyle may hold for blacks.

## Methods

### Recruitment

We recruited participants in AHS-2 through their churches and used a somewhat different approach for blacks than for whites. We based this decision on significant qualitative exploration, on pilot-study results comparing black and white recruitment with the same and differential approaches, and on past research indicating a general reluctance by blacks to participate in research studies (26-28). Local volunteers trained by AHS-2 staff recruited black church members through personalized, one-on-one contact at church. White participants received enrollment cards at church or through the mail. Promotional messages were placed in Adventist news media, both on television and in periodicals (26). Recruitment was voluntary, and we set goals on the basis of pilot-study results indicating that we could expect 65% of whites and slightly more than 50% of blacks who initially indicated interest to participate. Thus far, these expectations have been reasonably accurate, although the response for whites has been slightly lower than expected (62%), and that for blacks, slightly higher (52%). Recruitment for respondents described in this paper took place during 2001 to 2005.

Respondents were eligible if they were proficient in English and were aged 30 years or older for black respondents and 35 years or older for white respondents. The lower age cutoff for blacks acknowledges that their average age of onset of chronic disease is earlier than that for whites and reflects projected realistic target recruitment numbers in the available population. All instruments and procedures were approved by the Loma Linda University Institutional Review Board (IRB) in June 2001; approval was renewed annually thereafter.

### Questionnaire

Once enrolled, each participant received a previously validated (29,30) questionnaire and informed consent materials. We pilot tested the questionnaire with both black and white respondents for readability, comprehension, and relevance. To ensure adequate representation of foods more commonly eaten by blacks, we conducted dietary food frequency and validation studies (31). The questionnaire was divided into sections on disease and medication history, frequency of consumption of various foods, vitamin and mineral supplementation, physical activity, and other lifestyle practices. We asked respondents to inspect their pantries to verify the product and brand names of supplements, meat substitutes, and cereals.

The informed-consent materials briefly described the questionnaire and explained that the longitudinal nature of the study design would require annual follow-up surveys on hospitalization and other future data collection. We asked participants to give signed permission for their records to be linked with state cancer registries in the future and for hospitals to provide records in the event of a participant's death.

Questionnaires were returned by mail and edited for missing data and stray marks. We collected missing information by telephone when necessary and then converted data from the questionnaires into electronic files with an optical scanner, used SQL Server 2005 (Microsoft Corporation, Redmond, Wash) to merge this information, and analyzed the data with SAS version 8.0 (SAS Institute Inc, Cary, NC).

### Demographic measures

Respondents indicated their race and ethnicity by checking any of the following categories on the questionnaire: black/African American, West Indian/Caribbean, African, other black, Hispanic, and non-Hispanic white. Few respondents (N = 8) checked both a black category and the Hispanic category. Because this small number did not allow for meaningful analysis, we excluded these respondents. Participants were identified as white if they identified themselves as non-Hispanic white. Of the 56,754 participants in the study, 14,376 (approximately 25%) were non-Hispanic blacks (blacks) and 42,378 (approximately 75%) were non-Hispanic whites (whites).

Respondents reported their actual birth dates, which were then transferred into a continuous age measure and grouped into two categories: aged 30 to 59 years and aged 60 years or older. To indicate marital status, participants chose from six categories: never married, first married, remarried, common-law married, separated, divorced, or widowed. We recoded these data into currently married, never married, separated or divorced, and widowed. Respondents chose from the following options to report their level of education: grade school, some high school, high school diploma, some college, associate's degree, bachelor's degree, master's degree, and doctoral degree. We recoded these data into three categories: less than a high school diploma, high school diploma but less than a bachelor's degree, and bachelor's degree or higher. Respondents reported personal and household income by checking one of eight income categories, ranging from less than $10,000 to more than $200,000. We grouped these data into three income categories, separately for men and women: $20,000 or less, $21,000 to $50,000, and $51,000 or more [*sic*]. We also asked respondents whether they had worked for pay during the last year, and, if so, for how many hours. A variable for indicating whether respondents currently worked for pay (yes/no) and one for indicating how many hours they worked each week allowed us to determine the status of respondents who reported being retired but also indicated working for pay.

### Lifestyle measures

We asked participants to classify their overall health as excellent, good, fair, or poor and to report lifestyle practices related to smoking, alcohol use, meat and water consumption, physical activity, sleeping, and watching television.

To report smoking and alcohol use, participants chose from a series of questions about their history with these substances and then recorded a start and quit date for each as appropriate. We recoded responses into three categories for each substance: never, past use, and current use.

For meat consumption, participants reported whether they consumed items in any of the following categories: hamburger, ground beef; processed beef, lamb; beef or lamb as a main dish; pork, bacon; processed chicken, turkey; chicken or turkey roasted, stewed, broiled, fried; and fish. Frequency was indicated by choosing one of seven categories ranging from never or rarely to 2 or more times per day. We recoded responses into four categories: never, less than once per week, 1 to 4 times per week, and 5 or more times per week. For water consumption, we asked whether participants drank water, including sparkling water, but not counting coffee or tea. We recoded responses into three categories: less than 2 cups per day, 2 to 3 cups per day, and 4 or more cups per day.

We separated questions on physical activity into three intensity levels: low, medium, and high, with many examples of each listed. Participants reported the amount of time they spent in each type of activity during a normal weekday and on Saturday and Sunday. We totaled each participant's responses within each intensity category and then, calculating for men and women separately, used the totals to determine how many participants averaged at least 20 minutes of vigorous or extremely vigorous activity per day (high level of activity), how many averaged at least 20 minutes of moderate intensity activity per day (medium level of activity), and how many had lesser levels of activity (low level of activity). We calculated BMI (measured as weight in kilograms divided by height in meters squared [kg/m^2^]) from respondents' current weight and height and grouped these into four categories: underweight, BMI lower than 18.5; normal weight, BMI of 18.5 to 24.9; overweight, BMI of 25.0 to 29.9; and obese, BMI of 30 or higher.

Participants reported the amount of time they usually slept per night and the average number of hours per day they spent watching television in 1-hour intervals. We recoded responses on sleep into three categories: 6 or fewer hours, 7 to 8 hours, and 9 or more hours. We also recoded responses on watching television into three categories: 1 hour or less, 2 to 4 hours, and 5 or more hours.

### Self-reported diagnosed diseases and conditions

We asked if respondents had ever been diagnosed with a number of conditions, including high blood pressure, heart attack, high cholesterol, stroke, type 2 diabetes, and emphysema. Participants also answered a series of questions on whether a physician had ever told them that they had any form of cancer. Those who said yes then gave the year of first diagnosis for each of the cancer sites. Although we assessed a time frame for these diagnoses, we reported only whether the disease or condition had ever occurred.

### Data analyses

Data analyses include age- and sex-adjusted descriptive frequencies by race and ethnicity. To compare the prevalence of cancers (excluding skin cancers) between black and white participants, we used a random stratified sample of 320 black (D_1i_) respondents (141 men, 179 women) and 400 white (D_0i_) respondents (200 men, 200 women) who had reported having cancer. We calculated the proportion of non-skin cancers, q, in each age (5 strata), sex, and race category. For the total of black (N_1i_) and white (N_0i_) participants in the i^th^ age-sex stratum, a_i_ represents blacks diagnosed with cancer and b_i_, whites diagnosed with cancer; c_i_ represents blacks without a cancer diagnosis and d_i_, whites without a cancer diagnosis. Then for the i^th^ stratum, if PR is the prevalence ratio for stratum i,

log (PR_i_) = log {[(a_i_.q_li_)/N_li_]/[(b_i_.q_0i_)/N_0i_]},

which has the approximate variance

c_i_/(a_i_.N_1i_) + d_i_/(b_i_.N_0i_) + (1-q_l*i*_)/(D_1i_.q_*il*_) + (1-q*_0i_*)/(D_0i_.q*^0i^*).

The stratum-specific logs for the prevalence ratio are summarized as their weighted average, in which the weights are the inverse variances of the stratum estimates, and the variance of the summary statistic is calculated as usual (32). Hypothesis testing assumes that the summary log for the prevalence ratio is approximately normally distributed.

To test whether exposure variables with multiple categories differed between blacks and whites, we used logistic regression (one for each exposure) with ethnicity as the binary dependent variable. We used dummy-coded independent variables to represent age, sex, and the exposure of interest. To test the null hypothesis that the exposure distribution did not differ between black and white participants, we used a chi-square test to compare the full model with one without dummy exposure variables.

## Results

Blacks differed significantly (*P *<.001) from whites in sex (more were female), age (they were younger), marital status (fewer were currently married and more were never married, divorced or separated, or widowed), education (they were less highly educated) (Table 1). Personal income was lower for black than for white men (*P *<.001), but household income for the two groups did not differ significantly. Black women reported earning more than white women in personal income but having less than white women in household income. Also, more blacks than whites (73% v 58%) currently worked for pay, and blacks worked more hours per week (*P *<.001). Additional analyses not shown in the table indicate that black women were more likely than black men to have never married, to be divorced or separated, and not to be currently married. Because of the observed age and sex differences between black and white respondents and the well-known correlation of these variables with health, we adjusted all subsequent analyses for age and sex.

More black than white respondents reported having smoked in the past (*P *<.001), although current smoking rates were low for both racial groups (Table 2). A higher proportion of blacks than whites also reported having ever used alcohol (*P *<.001), but current use was lower for blacks than for whites (5% vs 7%, *P *<.001). Fewer blacks than whites (24% vs 48%) were vegetarian, and 35% of blacks reported consuming meat more than 4 days a week, whereas only 18% of whites reported this behavior (*P *<.001). Levels of water consumption were significantly lower for blacks than for whites (*P *<.001).

Overall, activity levels were higher for whites than for blacks (*P *<.001), and more blacks than whites (29% vs 21% of women, 25% vs 18% of men) reported low levels of activity. Although differences in levels of vigorous activity between blacks and whites did not differ significantly, men of both ethnicities reported higher levels of activity than did women. Finally, the two racial groups had significantly different BMI levels (*P *<.001). Forty-two percent of whites had normal weight according to their BMI; 28% of blacks were of normal weight. Obesity was more prevalent among blacks than among whites (35% vs 22%).

Blacks slept significantly less than did whites: 53% of blacks and 24% of whites slept 6 or fewer hours per night (*P *<.001). Blacks also reported spending more hours watching television. Overall, blacks ranked their health significantly lower than did whites *P *<.001): 15% of blacks and 26% of whites indicated excellent health; 21% of blacks and 12% of whites indicated fair health.

For self-reported prevalence of doctor-diagnosed diseases and conditions (Table 3), significantly more blacks than whites (*P *<.001) reported having a doctor's diagnosis of high blood pressure, stroke, and type 2 diabetes. Prevalences of myocardial infarction, high serum cholesterol, emphysema, and all cancers were lower for blacks than for whites (*P *<.001).

To further explore the substantially lower overall cancer results for black Adventists, we excluded skin cancers from the analysis and found that the lower risk remained for black women (*P *<.001), but not for black men (Table 4). Clearly, skin cancer was much less frequent among blacks than among whites in our study. The prevalence of prostate cancer, however, was much higher for black than for white men (OR = 1.47, *P *<.02).

Data not displayed in the tables show that blacks tend to become Adventists at a later age than do whites: the average age of baptism was 24.3 years for blacks and 21.2 years for whites (*P *<.001). Blacks were less likely than whites to identify themselves as Adventists at 15 to 25 years of age (*P *<.001), and black parents were less likely than white parents to have been Adventists when their children were aged 0 to 15 years (*P *<.001).

## Discussion

Our analyses allowed us to demonstrate some striking contrasts between white and black Adventists that warrant further exploration. The finding of shorter sleep duration for blacks is one of these differences. Other studies have found similar results by race for women (33) and evidence of different sleep physiology between the two racial groups (34). The link between sleep and obesity also warrants further examination. Despite our finding that the habits of black Adventists were somewhat less healthful than those of their white counterparts, their health habits on available comparison measures were considerably better than those of non-Adventist blacks: rates of smoking, drinking, and meat consumption were lower, and rates of vegetarianism and water consumption were higher (35).

The differences between black and white Adventists in self-reported disease prevalence are in line with data from other sources. Prevalences of hypertension and diabetes are known to be higher for blacks than for whites. That these disease rates are lower in our study than the comparable national rates for both blacks and whites, with the most strikingly lower rates being for black men, is noteworthy (36,37). More importantly, against expectations from the national data, the self-reported prevalence of diagnosed high serum cholesterol, emphysema, and cancer (except prostate) was actually lower for black than for white Adventists (38-40).

Although black respondents reported less healthy lifestyle practices overall than did whites, their low rates of current tobacco and alcohol use were similar to those of whites. The tendency of blacks to become Adventists at a later age than do whites may contribute to their longer lifetime use of both substances. Another possible explanation for the lifestyle differences between the two groups is that a larger proportion of white respondents are multigenerational Adventists and have a longer history of adherence to, and greater cultural support for, the recommendations of the church. Even though lifestyle behaviors were poorer for blacks than for whites in this cohort, their low current rates of smoking and alcohol use and their lifestyle behaviors, which are generally better than those of the total U.S. black population, may help explain why their health outcomes were better than those reported for blacks nationally.

Although these data should be interpreted cautiously, the power implicit in the large sample size used in the analyses of this study is strong. These results are encouraging but suggest a need for further study. For instance, the importance of spirituality in black U.S. culture and its known value as a disease mediator (41, 42) raise the question of whether this life component somehow mediates stress more for these Adventists and contributes to our finding that prevalences of a number of chronic diseases were lower for black than for white Adventists.

A number of study limitations must be considered when interpreting these findings. First, the data are self-reported, and health risk behaviors and disease states may be underreported. Second, observed differences in health outcomes may result at least partially from underdiagnosis among black respondents. Evaluation and treatment are often less aggressive for blacks than for whites with similar signs and symptoms, leading many to argue that health care system barriers and racism are behind underdiagnoses and late diagnoses, which are likely to result in poorer health outcomes (43). This discrepancy in care is unlikely to account entirely for the observed differences, however, given that the education level was higher for this black cohort (35% bachelor's degree or higher) than for blacks nationally (15% bachelor's degree or higher) (44).

Also, national data indicate that 80.3% of blacks and 88.7% of whites have health insurance (45). Although we do not have insurance data for the study population, national rates are most likely lower than those of this black cohort, whose education and full-time employment levels are higher than those for blacks nationally. Again, these facts may diminish the chances that all or most of the observed differences are accounted for by underdiagnosing alone. On the other hand, studies have found that blacks have less dyslipidemia, consistently higher HDL cholesterol, and sometimes lower LDL cholesterol than do whites (46,47). Published data on the prevalence of myocardial infarction among blacks are scarce. If the incidence of myocardial infarction is similar for blacks and whites, the explanation may be that mortality from myocardial infarction is higher for blacks and, thus, prevalence is lower.

Although the overrepresentation of women in our study should be noted as a concern, church-going populations usually have more women than men (48). The more pronounced difference for blacks is not surprising, given that church life plays a more central role in black than in white culture (49). The younger age of black respondents, although a result of eligibility requirements, should be noted as a potential study limitation even though all analyses were age-adjusted. Although we found that personal income was higher for black than for white women, we did not find this difference between black and white men. The reason may be that black women continue to work at later ages than do white women and are more often single heads-of-households, possibilities that are supported by the significantly lower household incomes among black than among white women. Exploring such complex issues as whether the stronger health benefits we observed for black men as compared with black women might be associated with added stress in black women's lives will be of interest. A final source of potential bias is the differential recruitment of black and white respondents to the study. We believe, however, that the need to recruit successfully far outweighs concerns about not using the same protocol for the two racial groups, a practice that has resulted in numbers too low for meaningful analyses in other studies.

The numbers in our study were large enough to allow us to explore whether the health benefits of lifestyle practices observed for white Adventists hold for black Adventists (7). Although the results for hypertension and diabetes were poorer for blacks than for whites, health outcomes in a number of categories were actually better for black than for white Adventists and especially better for black Adventists than for blacks nationally. With a full enrollment goal for AHS-2 of approximately 100,000 participants, 30,000 of whom are black, we will have adequate numbers to further investigate the hypothesis that black participants benefit even more than whites from a healthy diet and a tobacco-free, active lifestyle.

For these analyses, we plan to examine the independent effects of components of the Adventist lifestyle and their relative effects in these two groups by looking at high and low adherers to each lifestyle practice. We will also attempt to separate effects among blacks of Caribbean descent and those of American descent. This analysis will be limited, however, because blacks of Caribbean descent make up only 15% of the black study population, and not all endpoints are represented in this small group. Finally, because this cohort is recruited through churches, exploration of the role of spirituality as either a supplementary or a confounding factor is particularly relevant, and a separately funded study is already exploring this issue.

The potential contribution of our current and future findings to the national dialogue on health disparities is substantial. Evidence from this study acknowledges that black Adventists have a broad range of lifestyles, but just as the profile of health habits for white Adventists is better than that of white non-Adventists, the profile for black Adventists is better than that of their non-Adventist counterparts. Given the challenges of many other contributors to health disparities, including racism, housing segregation, employment discrimination, limited educational opportunity, and poorer health care, the study of the relative advantages of the Adventist lifestyle for blacks holds promise for helping to close the gap in health status between blacks and whites nationally.

## Figures and Tables

**Table 1:** Demographic Characteristics^a^ of Non-Hispanic Black and Non-Hispanic White Respondents (N = 56,754) to the Adventist Health Study-2, Preliminary Analysis, United States, 2001–2005

Demographic Characteristic	Non-Hispanic Black (N = 14,376), No.^b^ (%)^c^	Non-Hispanic White (N = 42,378), No.^b^ (%) c	***P* value^d^**
**Sex**			
Men	4,218 (29)	15,648 (37)	<.001
Women	10,158 (71)	26,730 (63)	
**Age, y**			
30-59	9,374 (66)	18,163 (43)	<.001
≥60	4,698 (34)	23,380 (56)	
**Marital status**			
Currently married	8,178 (58)	31,859 (77)	<.001
Never married	1,826 (13)	1,482 (4)	
Separated or divorced	2,700 (19)	3,977 (10)	
Widowed	1,368 (10)	4,351 (10)	
**Education**			
<High school diploma	1,652 (12)	2,798 (7)	<.001
High school diploma but <bachelor's degree	7,500 (53)	22,968 (55)	
≥Bachelor's degree	4,912 (35)	16,206 (38)	
**Annual personal income^e^**			
Men			
≤$20,000	1,094 (28)	3,762 (25)	<001
$21,000-$50,000	1,866 (48)	6,676 (45)	
≥$51,000	963 (25)	4,351 (29)	
Women			
≤$20,000	3,896 (43)	13,689 (57)	<.001
$21,000-$50,000	3,751 (41)	7,960 (33)	
≥$51,000	1,395 (15)	2,268 (9)	
**Annual household income^e^**			
Men			
<$20,000	651 (22)	2,713 (22)	<.001
$21,000-$50,000	1,015 (34)	4,371 (35)	
≥$51,000	1,343 (45)	5,367 (43)	
Women			
<$20,000	1,492 (25)	3,277 (17)	<.001
$21,000-$50,000	2,123 (36)	7,508 (40)	
≥$51,000	2,253 (38)	8,163 (43)	
**Currently working for pay**			
Yes	10,058 (73)	23,768 (58)	<.001
No	3,712 (27)	17,557 (42)	
**Hours worked/week^f^**			
≤20	949 (10)	3,869 (17)	<.001
21-50	7,551 (77)	15,616 (68)	
≥50	1,334 (14)	3,497 (15)	

a Adjusted for age and sex to account for observed differences in these variables between black and white respondents and the correlation of these variables with health. Reference group: non-Hispanic whites.

b Because of missing data, not all categories add to the total number.

c Because of rounding, not all categories add to 100%.

d Determined by chi-square test for independence.

e Income categories are as they appeared on the questionnaire.

f Retirees not included.

**Table 2:** Perceived Health Status^a^ and Selected Lifestyle Behaviors^a^ of Non-Hispanic Black and Non-Hispanic White Respondents (N = 56,754) to the Adventist Health Study-2, Preliminary Analysis, United States, 2001–2005

Health Status, Lifestyle Behavior	Non-Hispanic Black (N = 14,376), No.^b^ (%)^c^	Non-Hispanic White (N = 42,378), No.^b^ (%)^c^	***P *value^d^**
**Perceived health status**			
Excellent	2,089 (15)	10,575 (26)	<.001
Good	8,478 (62)	24,771 (61)	
Fair	2,836 (21)	4,963 (12)	
Poor	268 (2)	552 (1)	
**Smoking**			
Never	11,006 (78)	33,711 (80)	<.001
Past use	2,918 (21)	8,090 (19)	
Current use	240 (2)	332 (1)	
**Alcohol use**			
Never	7,703 (55)	25,634 (61)	<.001
Past use	5,745 (41)	13,663 (32)	
Current use	669 (5)	2,802 (7)	
**Meat consumption (times/week)**			
Never	3,431 (24)	20,463 (48)	<.001
<1	607 (4)	2,503 (6)	
1-4	5,341 (37)	11,996 (28)	
≥5	5,037 (35)	7,471 (18)	
**Water consumption (cups/day)**			
<2	2,557 (19)	3,991 (10)	<.001
2-3	2,893 (21)	7,703 (19)	
≥4	8,086 (60)	29,161 (71)	
**Physical activity (level/day)^e^**			
Women			
Low	2,957 (29)	5,533 (21)	<.001
Medium	3,210 (32)	10,638 (40)	
High	3,992 (39)	10,558 (40)	
Men			
Low	1,071 (25)	2,785 (18)	<.001
Medium	936 (22)	4,788 (31)	
High	2,210 (52)	8,074 (52)	
**Body mass index^f^**	
Underweight	192 (1)	871 (2)	<.001
Normal weight	3,718 (28)	16,907 (42)	
Overweight	4,927 (36)	14,051 (35)	
Obese	4,662 (35)	8,865 (22)	
**Sleep (hours/night)**			
≤6	7,232 (53)	9,841 (24)	<.001
7-8	5,931 (44)	28,439 (69)	
≥9	439 (3)	3,017 (7)	
**Television viewing (hours/day)**			
≤1	4,251 (31)	18,586 (45)	<.001
2-4	8,204 (60)	20,701 (50)	
≥5	1,150 (8)	1,944 (5)	

a Adjusted for age and sex to account for observed differences in these variables between black and white respondents and the correlation of these variables with health. Reference group: non Hispanic whites.

b Because of missing data, not all categories add to the total number.

c Because of rounding, not all categories add to 100%.

d Determined by chi-square test for independence.

e Low: <20 minutes of non-vigorous activity; medium: ≥20 minutes of non-vigorous activity; high: ≥20 minutes of vigorous activity.

f Underweight = BMI (kg/m^2^) <18.5; normal weight = BMI 18.5-24.9; overweight = BMI 25.0-29.9; obese = BMI ≥30.

**Table 3:** Age-Adjusted Prevalence^a^ of Self-reported Diagnosed Diseases and Conditions for Non-Hispanic Black and Non-Hispanic White Respondents (N = 56,754) to the Adventist Health Study-2, by Sex, Preliminary Analysis, United States, 2001–2005

Disease or Condition	Non-Hispanic Black (N = 14,376)			Non-Hispanic White (N= 42,378)			***P* value^b^**

	Male No. (%)	Female No. (%)	Total No. (%)	Male No. (%)	Female No. (%)	Total No. (%)	
High blood pressure	1325 (31.9)	3,392 (34.3)	4,717 (33.6)	3,814 (25.0)	6,854 (26.0)	10,668 (25.4)	<.001
Myocardial infarction	107 (2.6)	173 (1.8)	280 (2.0)	845 (5.4)	598 (2.3)	1,443 (3.4)	<.001
High serum cholesterol	856 (20.6)	2,059 (20.8)	2,915 (20.7)	3,918 (25.2)	7,473 (28.3)	11,391 (27.2)	<.001
Stroke	47 (1.1)	117 (1.2)	164 (1.2)	214 (1.4)	264 (1.0)	478 (1.1)	<.001
Type 2 diabetes	418 (10.6)	1,116 (11.3)	1,534 (10.9)	1,181 (7.6)	1,762 (6.7)	2,943 (7.0)	<.001
Cancer	280 (6.9)	576 (5.9)	856 (6.2)	3,231 (21.0)	4,788 (18.3)	8,019 (19.3)	<.001
Emphysema	33 (0.8)	48 (0.5)	81 (0.5)	229 (1.5)	244 (0.9)	473 (1.1)	<.001

a Reference group: non-Hispanic whites.

b *P* value is for the null hypothesis of equality in prevalences for blacks and whites, adjusting for age and gender.

**Table 4:** Age-Adjusted Odds Ratios (OR)^a^ for Self-reported, Diagnosed Cancer for Non-Hispanic Black and Non-Hispanic White Respondents (N = 56,754) to the Adventist Health Study-2, by Sex, Preliminary Analysis, United States, 2001–2005

Type of Cancer	Male		Female	
	OR (95% CI)	***P *value**	OR (95% CI)	***P *value**
All cancers	0.50 (0.44-0.56)	.001	0.42 (0.38-0.45)	.001
Cancers excluding skin cancer	1.08 (0.87-1.33)	.001	0.72 (0.62-0.85)	.001
Prostate cancer	1.47 (1.06-2.00)	.02	N/A	N/A

CI indicates confidence interval.

a Adjusted to account for observed differences in age between black and white respondents and the correlation of this variable with health. Reference group: non-Hispanic whites.
