# Erratum, Vol. 4: Issue 3

**Published:** 2008-01-15

Because of an editing error, two references in the article "Comparing Self-reported Disease Outcomes, Diet, and Lifestyles in a National Cohort of Black and White Seventh-day Adventists" were cited incorrectly. The fourth paragraph of "Discussion" should cite references 41 and 42, not 49 and 50. Corrections were made on our Web site on July 23, 2007, and appear online at www.cdc.gov/pcd/issues/2007/jul/06_0103.htm. We regret any confusion or inconvenience this error may have caused.

